# P-785. Pooled Antibiotic Susceptibility Testing (P-AST) Reveals High Prevalence of Multidrug- Resistance in Pediatric UTI Which Was Underreported by Standard Isolate Susceptibility Testing

**DOI:** 10.1093/ofid/ofaf695.996

**Published:** 2026-01-11

**Authors:** Sejal M Bhavsar, Nisha Polavarapu, Emery D Haley, Natalie Luke, Mohit Mathur, Xiaofei Chen, Jim Havrilla, David A Baunoch, Kenneth V Lieberman

**Affiliations:** Hackensack University Medical Center, Hackensack, NJ; Hackensack University Medical Center, Hackensack, New Jersey; Pathnostics, Wyoming, Michigan; Pathnostics, Wyoming, Michigan; Pathnostics, Wyoming, Michigan; Pathnostics, Wyoming, Michigan; Pathnostics, Wyoming, Michigan; Pathnostics, Wyoming, Michigan; Hackensack University Medical Center, Hackensack, New Jersey

## Abstract

**Background:**

Pediatric urinary tract infections (UTIs) can result in both acute complications, such as pyelonephritis or urosepsis, and chronic health complications, including recurrent UTI and renal damage. Therefore, rapid diagnosis and appropriate treatment of pediatric UTIs are essential.

**Figure 1.**
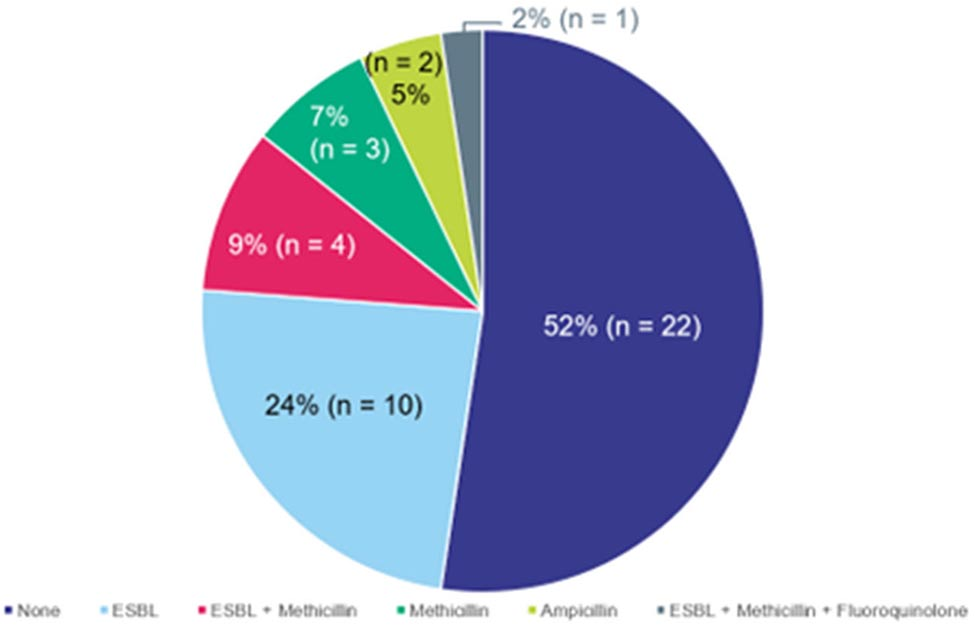
Frequency of Resistance Genes Detected

**Table 1.**
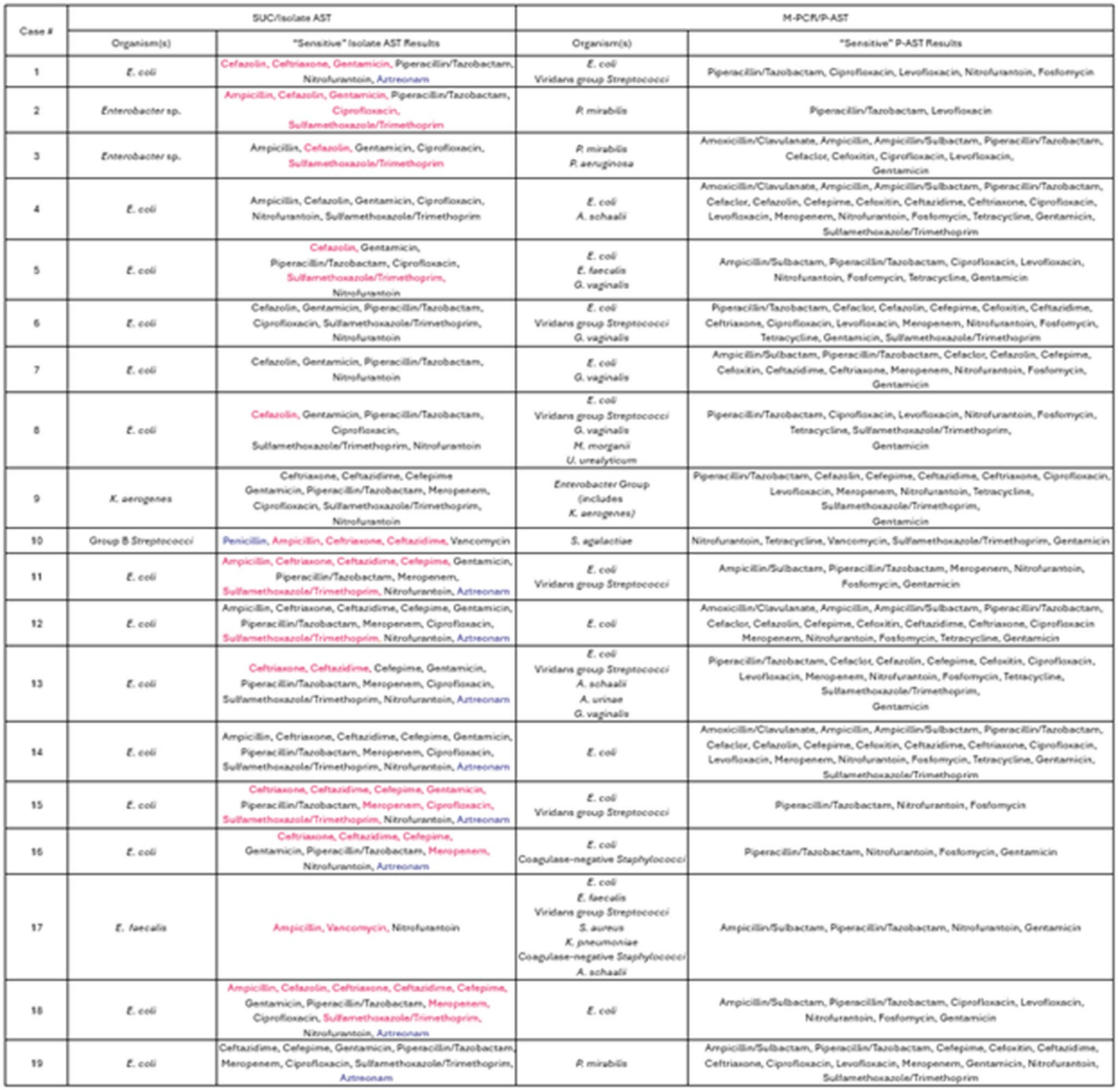
Side-by-Side Comparison of Antibiotic Susceptibility Results Between Standard Isolate Susceptibility Testing and P-AST Methods SUC = Standard Urine Culture; AST = Antibiotic Susceptibility Testing; M-PCR = Multiplex-Polymerase Chain Reaction; P-AST = Pooled Antibiotic Susceptibility Testing. Available SUC/Isolate AST results only included a list of antibiotics with “susceptible” results; P-AST reports included both lists of antibiotics with “susceptible” results and “resistant” results. Antibiotics listed in red were reported as susceptible by SUC/Isolate AST but as resistant by P-AST; Penicillin and Aztreonam (blue) were reported via SUC/Isolate AST but were not included in the P-AST assay.

**Methods:**

A previous study demonstrated superiority of Multiplex Polymerase Chain Reaction (M-PCR) over standard urine culture (SUC) in detecting microorganisms in the urine of 44 female and four male patients aged 3– 21 years old presenting to a Pediatric Emergency Department with clinically suspected UTI. Using the same cohort, this analysis explores the occurrence of phenotypic and/or molecular resistance detection by Pooled Antibiotic Susceptibility Testing (P-AST) and M-PCR in specimens with susceptible results from isolate testing.

**Results:**

M-PCR was positive for UTI pathogens in 42 specimens and P-AST was performed on 36 of these. Of the 36 tested by P-AST, resistance genes were also detected in 18 (Figure 1). The concordance between resistance gene detection and phenotypic resistance by P-AST was ≈ 60%. Two specimens that were positive for *S. aureus* had methicillin resistance genes detected, but both were negative for the colorimetric MRSA phenotype assay. No vancomycin or carbapenem resistance genes were detected. SUC was positive for UTI pathogens and reflexed to isolate susceptibility testing in 19 specimens, following the hospital standard protocol.

P-AST resulted 72% (n = 26) of cases as phenotypically multi-drug resistant (resistant to ≥ 1 antimicrobial agent from ≥ 3 classes). All available isolate AST results were compared to P-AST results and in 13 (68%) of these cases, P-AST reported resistance to antibiotics reported as sensitive by standard isolate testing (Table 1). P-AST is better able to detect heteroresistance and/or inter-species interaction effects in polymicrobial infections compared to isolate testing, which may explain these different results between methods.

**Conclusion:**

Resistance gene detection is insufficient to predict phenotypic antibiotic susceptibility. Antibiotic resistance was prevalent in pediatric UTI but was underreported by standard isolate susceptibility testing.

**Disclosures:**

Emery D. Haley, PhD, Pathnostics: Employee Natalie Luke, PhD, Pathnostics: Employee|Pathnostics: Stocks/Bonds (Private Company) Mohit Mathur, MD, PhD, Pathnostics: Employee|Pathnostics: Ownership Interest Xiaofei Chen, PhD, Pathnostics: ML algorithm|Pathnostics: employee Jim Havrilla, PhD, Pathnostics: ML algorithm|Pathnostics: Employee David A. Baunoch, Ph.D, Pathnostics: Have Patents on test with company but receive no compensation for it|Pathnostics: Ownership Interest

